# Nutrition support for critically ill patients during the COVID-19 pandemic: the Italian SIAARTI survey

**DOI:** 10.1186/s44158-022-00063-6

**Published:** 2022-08-09

**Authors:** Romano Tetamo, Ciro Fittipaldi, Salvatore Buono, Michele Umbrello

**Affiliations:** 1 Già Direttore UOC Anestesia E Rianimazione E Dipartimento Emergenza Urgenza, ARNAS Civico Palermo, Palermo, Italy; 2 UOC Anesthesia and Intensive Care, Hospital Pellegrini, Naples, Italy; 3 Direttore UOC Anestesia, Rianimazione E Terapia Intensiva, AORN Ospedali Dei Colli Presidio Ospedaliero CTO, Naples, Italy; 4 SC Anestesia e Rianimazione II, ASST Santi Paolo e Carlo – Polo Universitario, Ospedale San Carlo Borromeo, Milan, Italy

**Keywords:** COVID-19, Critical care, Nutritional support, Metabolism, Surveys and questionnaires

## Abstract

**Background:**

Critically ill, COVID-19 patients are characterized by a hypermetabolic state and a reduced food intake and are at high risk of malnutrition and lean body mass loss. An appropriate metabolic-nutritional intervention aims to reduce complications and improve the clinical outcomes. We conducted a cross-sectional, multicenter, observational, nationwide online survey involving Italian Intensivists to assess the nutritional practices in critically ill patients with COVID-19.

**Results:**

A group of experts in nutrition of the Italian Society of Anaesthesia Analgesia Resuscitation and Intensive Care (SIAARTI) developed a 24-item questionnaire; the 9000 members of the Society were invited to participate through emails and social networks. Data was collected from June 1 to August 1, 2021.

A total of 545 responses were collected: 56% in northern, 25% in central, and 20% in southern Italy. Artificial nutrition support is directly handled by intensivists in > 90 of the cases; the nutritional status is assessed as suggested by the guidelines in more than 70% of the cases, and a form of nutrition support is started within the first 48 h from ICU admission by > 90% of the respondents. Nutritional targets are reached in 4–7 days in > 75% of the cases, mainly by the enteral route. Indirect calorimetry, muscle ultrasound, and bioimpedance analysis are used by a limited part of the interviewees. Only about a half of the respondents reported the nutritional issues in the ICU discharge summary.

**Conclusions:**

This survey among Italian Intensivists during the COVID-19 epidemic showed how the beginning, progression, and route of nutritional support adhere to international recommendations, while recommendations on the tools to set the target and monitor the efficacy of the metabolic support are less followed.

**Supplementary Information:**

The online version contains supplementary material available at 10.1186/s44158-022-00063-6.

## Background

A complex cascade of metabolic and hormonal changes characterize critical illness and lead to hyperglycemia, lipolysis, and hypoaminoacidemia [1]. As compared to a classic population of critically ill patients, patients with coronavirus disease 2019 (COVID-19) are characterized by an even more marked hypermetabolic state, with elevated protein catabolism and energy expenditure, due to systemic inflammation, fever, and increased ventilatory work [2, 3]. As a consequence, critically ill, COVID-19 patients are exposed to a high risk of lean body mass loss and development of intensive care unit-acquired weakness (ICU-AW) [4, 5].

The COVID-19 pandemic has involved the healthcare systems on a global scale; in particular, ICUs have faced a completely unknown wave of patients. The disease primarily involves the respiratory system but may often lead to multiple organ failure and a significant mortality rate [6, 7]. Di Filippo et al. in their post hoc analysis of 213 COVID-19 patients reported a > 5% loss of the initial body weight in about one-third of the patients; notably, patients who lost weight had greater systemic inflammation, impaired renal function, and longer disease duration as compared with those who did not [8].

Malnutrition is very common in critically ill patients, and its development is a function of their pre-existing nutritional status and severity of illness (i.e., degree of hypermetabolism); moreover, admission to an ICU itself is associated with significant loss of lean mass, which in turn correlates with increased morbidity and mortality [9]. In addition, during the last decade, the critically ill patient population became older and their medical disorders more complex, with frequent comorbidities contributing to malnutrition. The COVID-19 pandemic exacerbates malnutrition: the incidence of malnutrition in patients hospitalized for COVID-19 reaches 50% [10, 11], and malnutrition was observed in mechanically ventilated patients even months after discharge from the ICU [12]. The wasting of muscle strength and mass has been termed sarcopenia [13]; from a pathophysiological standpoint, sarcopenia is considered a condition of muscle insufficiency [14], not dissimilar to how delirium is a form of brain insufficiency. It is only in the last years that Intensivists have become more familiar with this condition; indeed, a recent systematic review and meta-analysis found an average prevalence of sarcopenia of 40% of critically ill patients and a statistically significant correlation between the development of sarcopenia and an adverse outcome [15].

The examination of the nutritional status of critically ill patients at the beginning of hospitalization and early initiation of nutritional treatment are of great importance and essential to reduce complications and improve the clinical outcome both in terms of hospitalization and mortality [16]. Therefore, the prevention, diagnosis, and treatment of malnutrition must be considered a fundamental cornerstone of the therapy of the patient affected by COVID-19.

Starting from these clinical assumptions, the Italian Society of Anaesthesia Analgesia Resuscitation and Intensive Care (SIAARTI) conducted a survey inviting all the Italian intensivists members of the society to fill in a questionnaire to investigate the nutritional practices of COVID-19 patients. The aim of this survey was to provide insights into the opinions and beliefs of intensivists regarding the nutritional support in critically ill, COVID-19 patients. Secondary aims were to assess the possible association between the individual aspects of nutrition support and the age, geographical area, and type of hospital of the respondents.

## Methods

This is a cross-sectional, multicenter, observational, nationwide online survey involving Italian Intensivists, developed by SIAARTI. This article adheres to the appropriate Enhancing the QUAlity and Transparency Of health Research (EQUATOR) network reporting guideline, the Consensus-Based Checklist for Reporting of Survey Studies—CROSS [17].

The data gathered from survey respondents was completely anonymous with no personal information being collected and was not considered to be sensitive or confidential in nature. Therefore, ethical approval was not required for this type of study. Data were stored and handled in accordance with the interviewee’s privacy rights and following National privacy protection laws, and the survey software access was password-protected to prevent any unauthorized access to the data. Participants were asked for their written informed consent.

### Development of the questionnaire

A group of experts on nutrition in intensive care, designed by SIAARTI, was asked to develop the questionnaire. The structure of the survey was based on a preliminary analysis of the state of the art of the literature on this issue and on international guidelines; a member of the panel (SB) drafted a first version of the questionnaire and spread it to the other members. A modified Delphi technique was used, and ‘rounds’ were held until group consensus was reached. The final formulation of the questions was unanimously voted and approved by the board. The questionnaire was then built with SurveyMonkey Platinum (SurveyMonkey Inc., San Mateo, CA, USA).

To avoid multiple participation of respondents, a closed-structure survey was designed, in which the access to the questionnaire was protected by registration, and a unique, anonymous identifier was assigned to each respondent. The questionnaire consisted of 24 questions in total. A first part included questions concerning the age of the respondent and the region, type of hospital, and type of ICU in which he worked. The remaining part of the survey was divided into a section dealing with the nutritional risk screening, assessment of nutritional requirements and monitoring of the metabolic intervention, a section about the route of nutrition and time to start the support and to reach the targets, a section on the types and formulation of artificial support administered and a last section about the strategies to overcome enteral nutrition intolerance.

Questions were all mandatory to increase the completeness of the survey. Completion and internal consistency of all items was enforced using server-side techniques, i.e., displaying the questionnaire after submission and highlighting mandatory but unanswered items or items answered inconsistently. The range of responses was based on a Likert-like scale (*never*, *rarely*, *sometimes*, *often*, *all the time*) when appropriate, and all items provided a non-response option such as “*not applicable*” or “*rather not say.*” Log-file/IP address analysis was used to prevent a single user to fill in the same questionnaire multiple times. All answers could be reviewed and edited through a review step which displayed a summary of the responses and asked the respondents if they were correct until the final submission. Supplementary figure S1 shows an adapted English version of the online survey.

The survey included a cover letter which included information about the survey structure and its purpose, details of investigators, including contact details in case clarifications were desired, described data anonymization process, and asked for interviewee’s consent for participation and subsequent publication of the results.

### Population and sampling techniques

Invitations to participate were distributed to all the SIAARTI members by direct emails and through social media platforms (namely, Facebook, Instagram, Twitter, and LinkedIn). Data was collected from June 1 to August 1, 2021. In order to collect data representative of the national scenario, the panel of experts decided not to apply inclusion and exclusion criteria for the study. Collection was totally anonymous; all survey participants were included on a voluntary basis and received clear answers about the purpose of the survey and the use of the data collected.

A convenience sampling strategy was used. Considering a population of 9000 SIAARTI members, and an acceptable margin of error of 5%, we calculated that a sample size of at least 450 respondents would be required to have a 95% confidence level in the responses [18].

### Data Analysis

Only completed questionnaires were analyzed, so no missing data were present in the dataset. Data were downloaded as a “.csv” file and subsequently stored as a single-source Excel file (Microsoft Corp, Redmond, WA, USA), handled by only one member of the panel.

Continuous variables were summarized using the median and interquartile range, while categorical variables using counts and percentages. The presence of a possible association between the individual items under study and the aggregation variables (class of age of the respondents, geographical area, type of hospital) was assessed by a chi-square statistical test; appropriate corrections were applied for multiple comparison. *P*-values < 0.05 were considered statistically significant.

## Results

### Survey respondents

A total of 545 intensivists filled in the online survey, with a response rate of 6.1% of the total population of Italian SIAARTI members. Baseline characteristics are reported in Table 1. Among those who responded to the survey, 56% came from northern, 25% central, and 20% southern Italy; general and teaching Hospitals were the main institutions in which the interviewee worked, and the ICUs were mainly general or COVID-19-specific cohort units. Notably, nutrition support was managed by Intensivists in 92% of the cases, whereas only in the remaining 8% of the cases by a Clinical Nutrition specialist.

**Table 1:** Baseline characteristics of the questionnaire respondents

Variable	Respondents (*n* = 545)
Age (years)	43 ± 10
Years of experience, *n* (%)	
Resident, 1st year	8 (1%)
Resident, 2nd–3rd year	29 (5%)
Resident, 4th–5th year	43 (8%)
Attending, 1–10 years experience	185 (34%)
Attending, 11–20 years experience	180 (33%)
Attending, 21–30 years experience	63 (12%)
Attending, 31–40 years experience	31 (6%)
Attending, > 40 years experience	4 (1%)
Geographical area, *n* (%)	
Northern Italy	310 (57%)
Central Italy	130 (24%)
Southern Italy	103 (19%)
Type of hospital, *n* (%)	
General hospital	286 (53%)
Teaching hospital	173 (32%)
Private research hospital	24 (4%)
Public research hospital	23 (4%)
Religious hospital	11 (2%)
University-owned hospital	5 (1%)
Other	21 (4%)
Type of ICU, *n* (%)	
General ICU	362 (67%)
COVID-19 cohort ICU	107 (20%)
Post-operative ICU	28 (5%)
Cardiac ICU	31 (6%)
Neuro ICU	11 (2%)
Pediatric ICU	4 (1%)

### Nutritional risk, nutritional requirements, and monitoring of the metabolic intervention

Nutritional risk screening is mainly assessed as suggested by European Society for Clinical Nutrition and Metabolism (ESPEN) guidelines (71% of respondents), whereas tools as the Nutritional Risk Screening (NRS) 2002 and Nutrition Risk in the Critically Ill (NUTRIC) Score are less used (41%); body weight and body-mass index (BMI) are used by > 80% of the interviewees.

Monitoring of nutritional status is performed by the measurement of urine nitrogen excretion and the calculation of the nitrogen balance by about 2/3 of the respondents; ultrasonographic assessment of lean body mass is used in 14% of the cases, bioimpedance analysis is used by 3% of the respondents, while 13% of the interviewees do not use any type of monitoring of the metabolic intervention. Nitrogen balance is performed on a daily basis for the whole duration of ICU stay in 13% of the cases, in 1% on a daily basis only for the first week, in 50% of the cases it is calculated less than daily and it is not assessed by 35% of the interviewees.

The calorie target is calculated following ESPEN guidelines in 81% of the cases; equations are used by 46% of the respondents, whereas indirect calorimetry is used only by 21% of the respondents. Functional assessment of patient status is performed with validated scales in 17% of the cases.

**Fig. 1:**
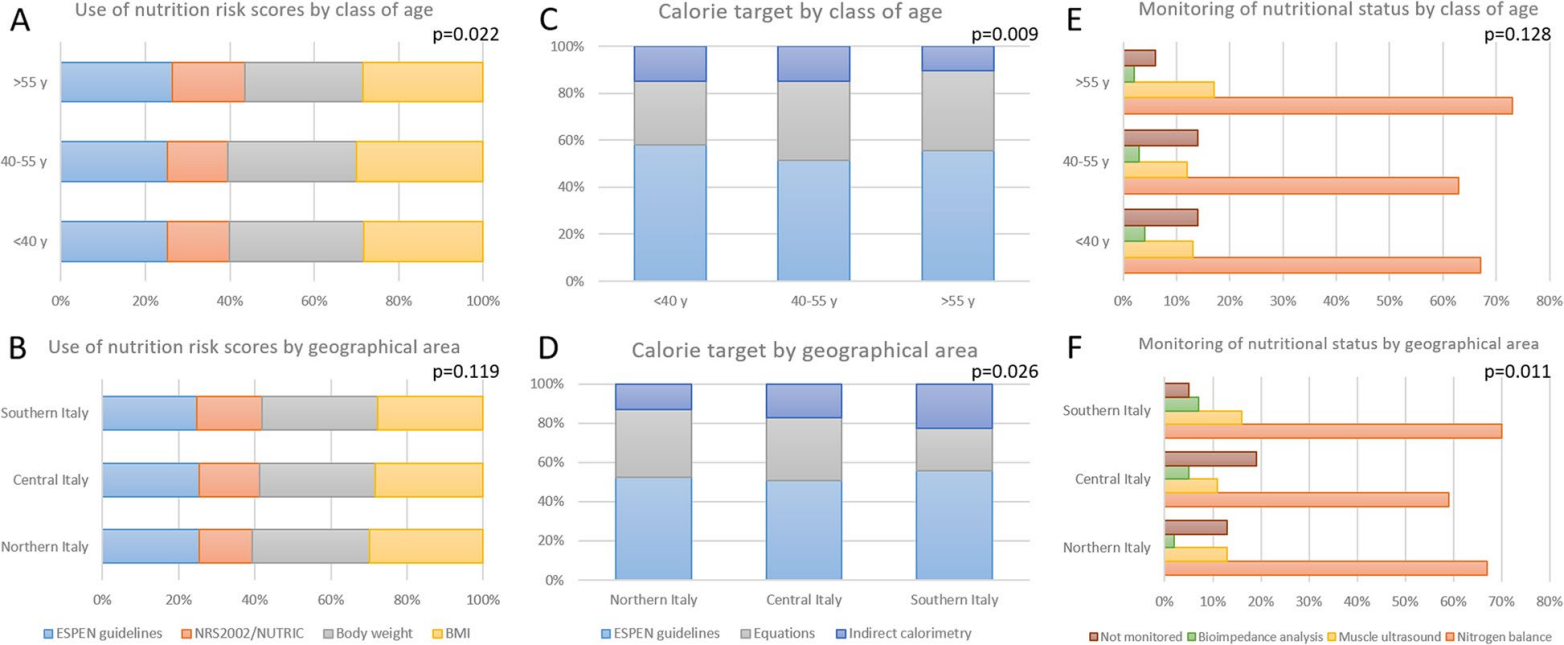
Relationship between nutritional risk scores (left panel), nutritional requirements (middle panel), and monitoring of the metabolic intervention (right panel) and the class of age (upper) and geographical area (lower) of the respondents

Figure 1 shows the relationship between nutritional risk scores, nutritional requirements, and monitoring of the metabolic intervention and the class of age and geographical area of the respondents; supplementary figure S2 reports the same data by the type of hospital of the respondents.

### Route of nutrition and time to start the support and reach the targets

Artificial nutrition is started within the first 24 h from the moment in which the patient is considered clinically stable by 53% of the interviewees, 25–48 h by 41%, and > 48 h by 6% of the respondents. The calorie target is reached in 4–7 days in 70%, between 7 and 14 days in 21%, and > 14 days in 9% of the cases; the time to reach the target is similar between COVID-19 and non-COVID-19 critically ill patients. As for the route of artificial nutrition, enteral nutrition is used by 65% of the interviewees, 4% use an exclusively parenteral nutrition, whereas 31% use a mixed enteral-parenteral support.

**Fig. 2:**
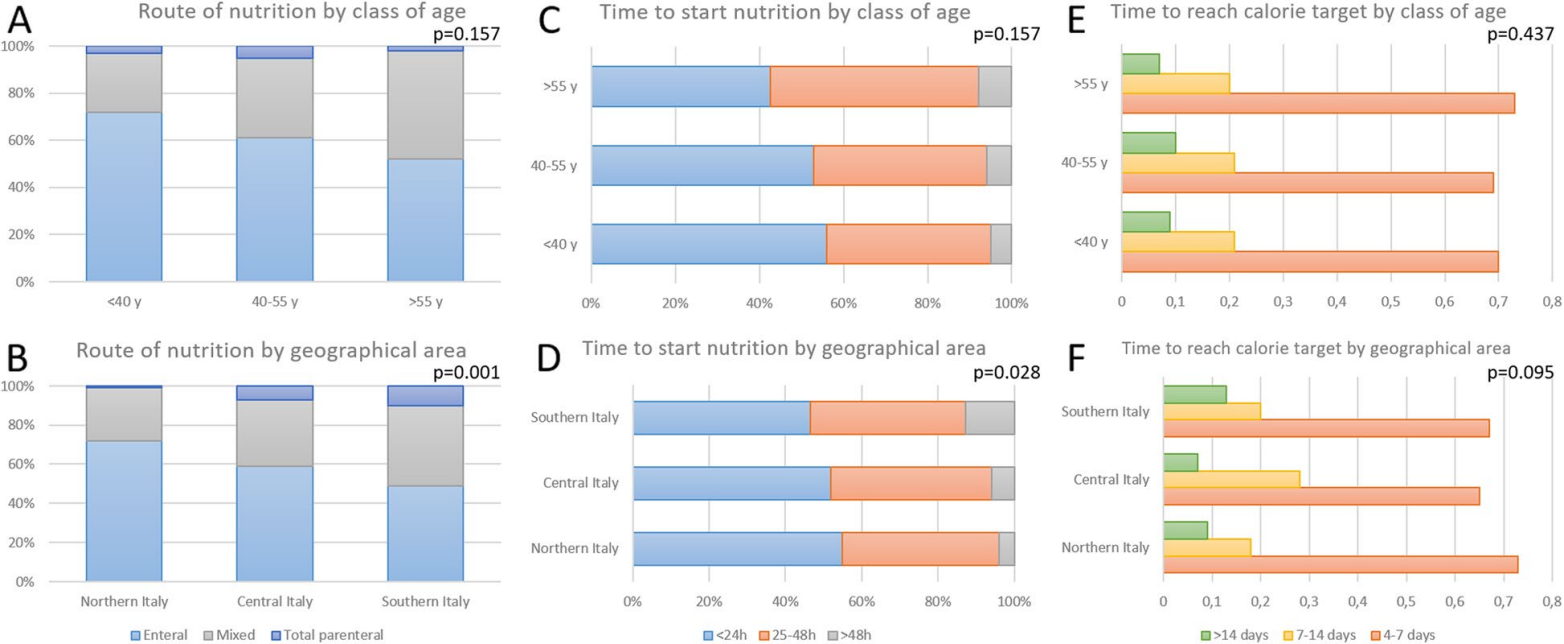
Relationship between the route of nutrition (left panel), time to start the support (middle panel), and time to reach the target (right panel) and the class of age (upper) and geographical area (lower) of the respondents

Figure 2 shows the relationship between the route of nutrition and time to start the support and to reach the targets and the class of age and geographical area of the respondents; supplementary figure S3 shows the same data by the type of hospital of the respondents.

### Types and formulation of artificial support administered

If an enteral nutrition was used, in 57% of the cases intensivists used a standard isocaloric formula, whereas in 34% of the cases an individualized formula was used; in the remaining 8% of the responses, no specific formula was indicated by the respondents. When parenteral nutrition support was delivered, a standard, commercially-available formulation was used in 48% of the cases, a special formulation was used in 22% of the cases, a formulation individually prescribed by a clinical nutrition specialist was used in 18% of the cases, while the remaining 5% of the respondents did not indicate the type of formulation used and 7% responded that no parenteral nutrition was ever delivered.

After the resolution of the acute phase of illness, in 55% of the cases patients were discharged from the ICU with oral nutritional supplements, 25% with ongoing enteral nutrition, and 1% with parenteral nutrition. In 20% of the cases, respondents did not indicate the type of prescription upon discharge. Only 51% of the interviewees reported the nutritional problems in the discharge summary.

**Fig. 3:**
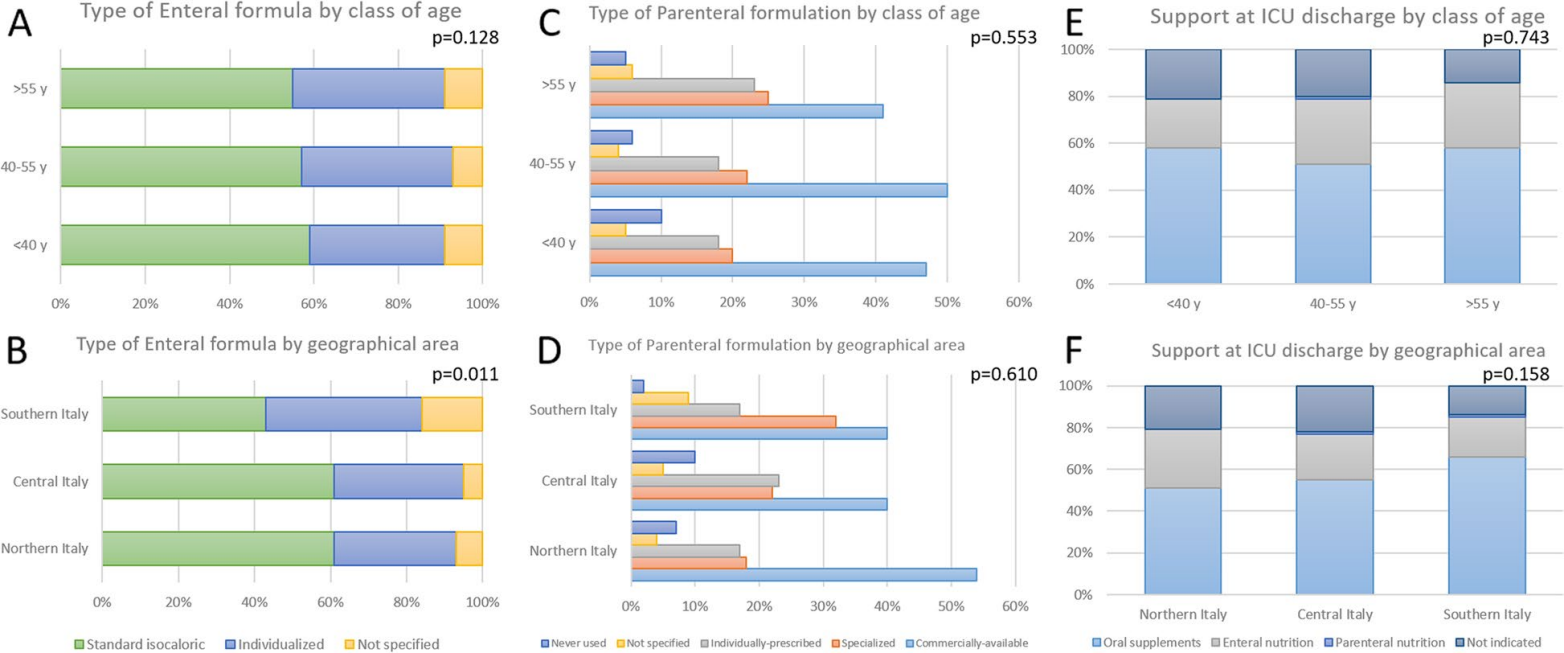
Relationship between the type of enteral formula (left panel), parenteral formulation (middle panel), and type of support at ICU discharge (right panel) and the class of age (upper) and geographical area (lower) of the respondents

Figure 3 shows the relationship between the types and formulation of artificial support administered and the class of age and geographical area of the respondents; supplementary figure S4 shows the relationship with the type of hospital of the respondents.

### Strategies to overcome enteral nutrition intolerance

In case of the development of enteral nutrition intolerance, 40% of the respondents use prokinetic drugs with a threshold for prescription influenced by the assessment of gastric residual volumes (GRVs), 31% use both prokinetic drugs and a supplementary parenteral nutrition, 16% only supplementary parenteral nutrition, 9% use prokinetic drugs independently of the level of GRVs, 2% switch to total parenteral nutrition, and 2% use a post-pyloric feeding approach.

**Fig. 4:**
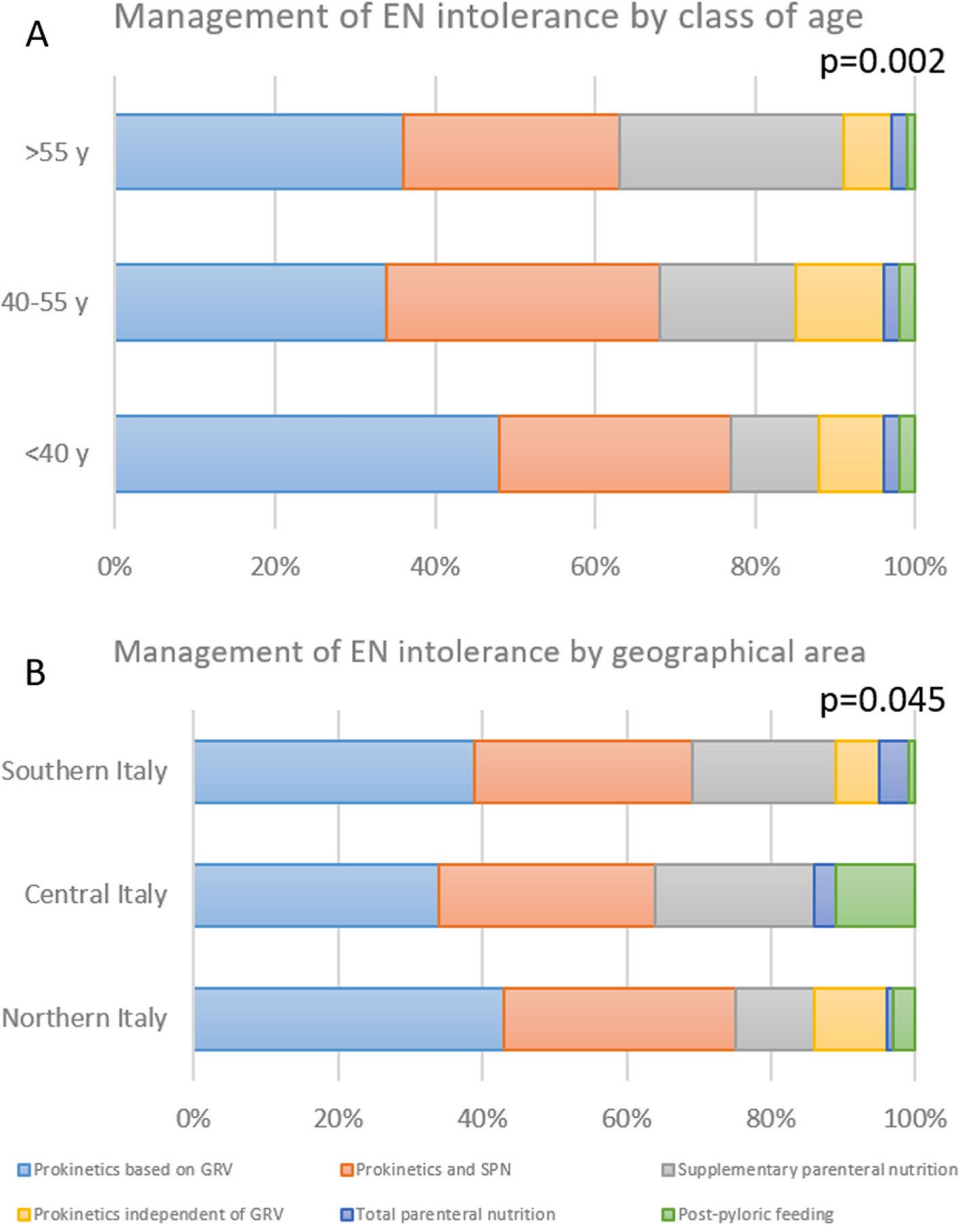
Relationship between the strategies to overcome enteral nutrition intolerance and the class of age (upper) and geographical area (lower) of the respondents

Figure 4 shows the relationship between the strategies to overcome enteral nutrition intolerance and the class of age and geographical area of the respondents; supplementary figure S5 shows the same data according to the type of Hospital of the respondents.

## Discussion

### Main findings

The main findings of this multicenter, national survey on the nutritional practices of Italian Intensivists during the COVID-19 pandemic can be summarized as follows: artificial nutrition support is directly handled by intensivists in > 90 of the cases; the nutritional status is assessed as suggested by the ESPEN guidelines in more than 70% of the cases, and a form of nutrition support is started within the first 48 h from ICU admission by > 90% of the respondents, with nutritional targets which are met in 4–7 days in more than 75% of the cases, and mainly by the enteral route. However, despite the international recommendations [19, 20], indirect calorimetry, muscle ultrasound, and bioimpedance analysis are underused to set the target and monitor the efficacy of the metabolic support. Eventually, only about a half of the respondents reported the nutritional issues in the ICU discharge summary.

While several scientific societies have updated their recommendations to guide the metabolic treatment of COVID-19 patients in the ICU [19–22], growing evidence indicates how these patients have a higher metabolism and yet receive a lower nutritional support than their non-COVID counterparts [23]. The data from this survey can then provide an insight into the clinical and organizational factors which might influence the administration of an adequate nutrition support in critically ill, COVID-19 patients.

### Basis of metabolic and nutrition support in critically ill patients with COVID-19

A severe respiratory infection such as COVID-19 induces an inflammatory syndrome and increases energy expenditure, which is in turn responsible for increased calorie and protein requirements. On the other side, food intake is reduced by infection- and stress-induced anorexia and dyspnea. As a consequence, most patients are at high risk of muscle wasting [24]. The prevention, diagnosis, and treatment of malnutrition is a key part of the approach to COVID-19 patients, as it aims to improve both short- and long-term outcomes. Sarcopenia should therefore be prevented by an appropriate metabolic support, including adequate nutrient delivery and stimulation of physical activity [20]. Critically ill patients with COVID-19 should be first screened for malnutrition with validated scales such as the NRS-2002, and then assessed for the diagnosis and grading the severity of malnutrition, according to the specific phenotypic and etiologic criteria [25].

### Assessment of calorie targets

Despite the recommendations [19–22], calorie requirements were mainly determined by the use of predictive equations, while indirect calorimetry, the gold standard for the measurement of energy expenditure [26], was limited to about 20% of the responses.

Indeed, predictive equations have proved inaccurate and poorly reproducible [27], potentially leading to up to 1000 kcal/day targets higher or lower than measured needs [28], thereby increasing the risk of under- or overfeeding. This limitation concerns not only the equations developed from healthy subjects (i.e., the Harris-Benedict equation), but also those specifically developed in critically ill patients, such as Penn-State equation [29].

On the other side, meta-analytic data showed a > 20% reduction in short-term mortality when calorie targets were based on calorimetry [30]. We did not collect data about the reason for not using indirect calorimetry. Among the organizational issues potentially related to the unavailability of the device, investment costs, consumables, calibration gas, service, and staff time should all be taken into account. Italian ICUs experienced a massive overload of COVID-19 patients, and this context might have precluded the use of any sophisticated, non-vital device. Moreover, some technical issues can at least partially explain its limited use [31]: in ventilated patients with FiO2 > 0.6 and PEEP > 12 cmH_2_O, no reliable measurements are possible, and these values are not dissimilar from the average data of COVID-19 patients [32]. Furthermore, the presence of air leaks such as pneumothorax or subcutaneous emphysema, preclude the measurement of energy expenditure, as not all exhaled gas pass through the device sensors. In COVID-19 patients, an unexpectedly high incidence of barotrauma has indeed been described [33].

Recently, an alternative to indirect calorimetry has been described and validated to calculate energy expenditure based on ventilator-derived carbon dioxide production as measured by the built-in capnometer of the mechanical ventilator [34]. Despite being criticized by some authors [35], this method was shown to correlate better with indirect calorimetry values than estimates derived from weight-based equations [36] and has the unique advantage of being easily available at the bedside.

### Monitoring of lean body mass

The critically ill patient loses a significant amount of proteins, to provide amino acids for endogenous substrate production and gluconeogenesis [37]. The gold standard for the evaluation of lean body mass distribution is CT scans at the lumbar region [38]; however, such measurements are seldom readily available.

Despite the efforts to reduce operator measurement error, anthropometric data such as body weight and BMI are generally unreliable in critically ill patients [39]. Moreover, they give no indication of body composition, muscle mass, or nutritional state. Additionally, inflammation makes serum proteins, including prealbumin, unreliable [40].

The loss of lean body mass, and the effects of a nutritional intervention, is generally evaluated by the assessment of urine nitrogen excretion; muscle mass can also be measured using bioimpedance or ultrasound. Despite the known limitations of nitrogen balance in critically ill patients [41], the current survey suggests how this is still the mainly used method in up to 2/3 of the respondents, whereas ultrasonography or bioimpedance are used in less than 20% of the cases in total.

However, the determination of nitrogen balance is prone to significant measurement errors, both as concerns the accurate determination of protein intake as well as of all sources of nitrogen excretion. The method used in clinical practice assumes that total nitrogen loss corresponds to urinary urea nitrogen excretion plus an additional constant [42]; such factor accounts for the part of nitrogen loss deriving from urinary non-ureic nitrogen loss, as well as skin, gastrointestinal and insensible losses. However, these assumptions underestimate non-ureic urinary nitrogen in catabolic critically ill patients [43]. Notably, nitrogen balance reflects only the net result of nitrogen exchange, while it does not provide any insight into the dynamics of protein synthesis, catabolism, or redistribution [41].

Bioelectric impedance analysis (BIA) is a quick, non-invasive, and relatively inexpensive technique to assess body composition, which is based upon the determination of the impedance of an electric current passing through the body [44]. The main drawback of such a method is that it assumes static ratios between the compartments, most notably a fixed hydration of tissues, which often does not apply to critically ill patients, making the data less reliable [45]. A recent investigation showed how in critically ill patients BIA-assessed body composition is significantly modified after one week of ICU stay, and how BIA may be useful to define the hydration state, while it does not seem to track muscle mass [46]. Moreover, in critically ill patients, the nutritional status assessed by ultrasonography, but not by BIA, was shown to predict 28-day mortality [47].

Muscle ultrasonography allows the direct visualization and classification of muscle mass and characteristics [48]; it can be performed at the bedside, is non-invasive and readily available, reliable, and can be used to detect changes in the trajectory of muscle mass quality and quantity [49], allowing for the assessment of the development of sarcopenia. Indeed, a recent systematic review and meta-analysis found a pooled prevalence of sarcopenia in hospitalized patients with COVID-19 of about 50% [50], and the duration of ICU stay is the main determinant of persistent sarcopenia at 3 months after recovery [51]. Interestingly, baseline muscle ultrasound characteristics in critically ill, COVID-19 survivors showed a significantly lower echointensity as compared with those who did not survive [52]. A prospective, single-center study in COVID-19 patients found that sonographic assessment of a low muscle mass was associated with an adverse outcome [53]. Eventually, early changes in muscle size and quality were found to be related to the outcome of critically ill COVID-19 patients and to be influenced by nutritional and fluid management strategies [54]. Given the widespread availability of ultrasound devices in ICUs, and the smooth learning curve of the technique, which can be acquired with an excellent reliability after a 2-day course [55], specific programs for the implementation of this technique are welcome in the next future.

### Management of enteral feeding intolerance

In case of intolerance to enteral nutrition, 80% of respondents used prokinetics, either conditional on the presence of gastric residual, independently of the level of GRVs, or together with supplementary parenteral nutrition (sPN). This is in accordance with the ESPEN guidelines [19], which recommend the use of sPN only in case of failure of all strategies aimed at optimizing gastric tolerance.

GRV has long been considered a surrogate parameter of gastrointestinal dysfunction and a routine part of enteral feeding protocols. However, the value of periodic GRV measurements, especially with regard to a reduction in the risk of pneumonia, has frequently been questioned in the past years [56], and little scientific evidence indicates that this improves patient outcomes [57]. Moreover, the practice of measuring GRV is neither standardized nor validated, and lacks reproducibility by several determinants, including patient-, tube, and technique-related variables. Indeed, about 50% of COVID-19, critically ill patients develop gastrointestinal hypomotility and enteral feeding intolerance [58], which is not solely explained by the effects of vasoconstriction from vasopressor or the use of sedatives and opiates and can possibly be related to some degree of gastrointestinal involvement specific to SARS-CoV-2 infection [59].

Notably, only 2% of the interviewees reported the use of post-pyloric feeding, while the European guidelines suggest its use in case of intolerance to enteral nutrition not solved with prokinetic agents [19, 20].

### Discharge from intensive care

Coordination and continuity of care, and especially the nutritional program, at the moment of ICU discharge, is a key component of a comprehensive strategy aiming to improve the functional outcome of patients. In the current survey, only about a half of the respondents reported information on the nutritional status and support in the discharge notes. This is not unexpected, as communication between ICU and general ward healthcare providers has been documented to be infrequent, incomplete, and of poor quality [60]. However, in a recent trial, energy and protein intake in the post-ICU hospitalization period were between 30 and 50% less than estimated and measured requirements, and oral nutrition provided alone was the most common mode of nutrition therapy [61].

In order to improve the outcome of critical illness, a multimodal intervention for an optimal nutrition therapy should be provided starting from the admission to an ICU and carried over until discharge home. Analogously to antimicrobial stewardship, nutrition stewardship programs, defined as the effort to optimize artificial nutrition use with the aim of improving patient outcomes, ensure cost-effective treatments, and reduce adverse sequelae [62], need to be organized and spread across the country.

### Strengths and limitations of this study

Online surveys enable the collection of anonymized information and facilitate the collection of data from a wide range of respondents regardless of their residence. The survey was disseminated through social media to reach a wide turnout. However, such an advertisement could have led to a skewed distribution of respondents and caused a selection bias, as physicians who do not have social media could not have filled in the survey.

The survey did not include any questions about the use of continuous vs. intermittent enteral feeding. International guidelines [19] recommend the use of continuous feeding rather than intermittent boluses due to the lower incidence of side effects such as diarrhea; however, despite a strong consensus the grade of the recommendation is low, mainly because of the limited sample size and heterogeneity of the populations included, and the lack of proven benefits in other outcome measures. Indeed, the debate about the best strategy to deliver enteral feeding has grown in the last years, based on the concept that an intermittent pattern could better mimic the normal daily life feeding pattern [63], and on reports that continuous enteral feeding might improve the achievement of target nutrition requirements as compared to an intermittent pattern [64]. A recent systematic review which included 19 studies that compared the two approaches found both results in favor of a continuous pattern (such as lower gastric residuals and less need for prokinetics), and in favor of an intermittent one (i.e., better digestive tract colonization, lower constipation and less evidence of tracheal aspiration of gastric contents), and confirmed that the current level of evidence is not sufficient to provide clear indications on which approach should be preferred [65].

## Conclusions

This survey among Italian Intensivists during the COVID-19 epidemic provided an insight into the nutritional practices provided to critically ill, COVID-19 patients. In particular, we found how the beginning, progression, and route of nutritional support adhere to international recommendations, while recommendations on the tools to set the target and monitor the efficacy of the metabolic support, as well as reporting the nutritional problems in the ICU discharge summary are less followed. These results can be the basis which educational and stewardship interventions aimed at increasing the awareness of Italian intensivists on this topic can be built upon, with the aim of improving the care of critically ill patients.

## Supplementary Information

**Additional file 1: Supplementary Figure S1.** English version of the survey.

**Additional file 2: Supplementary Figure S2.** Relationship between nutritional risk scores, nutritional requirements and monitoring of the metabolic intervention and the type of Hospital of the respondents. **Supplementary Figure S3. **Relationship between the route of nutrition and time to start the support and to reach the targets and the type of Hospital of the respondents. **Supplementary Figure S4.** Relationship between the types and formulation of artificial support administered and the type of Hospital of the respondents. **Supplementary Figure S5. **Relationship between the strategies to overcome enteral nutrition intolerance and the type of Hospital of the respondents.

## Data Availability

The datasets generated during and/or analyzed during the current study are available from the corresponding author on reasonable request.
