# Supplementary material for: Nutrition support for critically ill patients during the COVID-19 pandemic: the Italian SIAARTI survey
Source: J Anesth Analg Crit Care. 2022 Aug 9;2:35. doi: 10.1186/s44158-022-00063-6 (PMC9361260; doi:10.1186/s44158-022-00063-6)
Supplement: Supplementary file 1 — Additional file 1: Supplementary Figure S1. English version of the survey. [file 44158_2022_63_MOESM1_ESM.pdf]

SIAARTI

PRO VITA CONTRA DOLOREM SEMPER

Survey on the nutritional practices of critically ill, COVID-19 patients

For more than a year and a half, the SARS-Cov-2 pandemic has been giving a tremendous strain on the organizational and care resilience of health care systems around the world and, consequently, on the lives of millions of people.

Italy was the first Western country to be affected by the pandemic. In April 2020, during the first wave, more than 28,000 patients were admitted to Italian hospitals, and more than 3,500 of them were in intensive care units.

SARS-Cov-2 syndrome, which in about 10-15% of cases manifests as severe illness, in 5-7% of cases requires intensive care unit admission for severe respiratory failure.

The characteristics of patients admitted to the ICU (elderly, with comorbidities, malnourished), coupled with the severity of conditions resulting from SARS-Cov-2 syndrome, should lead clinicians to consider nutritional support as an integral part of the therapeutic approach in the ICU setting. The indication for nutritional therapy, in fact, aims for the prevention and modulation of the effects which critical illness causes on nutritional and metabolic status, as well as to alleviate the malnutrition status, often present in the majority of Covid-19 patients.

To make a contribution to this issue, SIAARTI is conducting an exploratory survey on the nutritional strategies of critically ill patients admitted to the ICU with severe forms of Covid-19; we aim to understand how the nutritional status of the critically ill patients is addressed by Italian intensivists.

1. The ICU in which you work has admitted pazients with COVID-19?

- ◯ Yes
- ◯ No

SIAARTI
PRO VITA CONTRA DOLOREM SEMPER

Survey on the nutritional practices of critically ill, COVID-19 patients

2. Insert your e-mail address:

* 3. What is your genre:

- M
- F
- Prefer not to say

* 4. What is your age:

* 5. When did you complete your post-graduate Anteshesia and Intensive Care program?

* 6. Which Region do you work in:

* 7. Which city o you work in:

* 8. Which type of Hospital do you work in

- General Hospital
- Teaching Hospital
- Private research Hospital
- Public research Hospital
- Religious Hospital
- University-owned Hospital
- Other (specify)

* 9. Write the name of the Hospital you work in:

* 10. What type of ICI do you work in

- General ICU
- COVID-19 cohort ICU
- Neuro-ICU
- Cardiac ICU
- Postoperative ICU
- Pediatric ICU

* 11. In your ICU, who is in charge for the management of nutrition support:

- Intensivist
- Clinical nutrition specialist

* 12. What tool for the nutritional screening do you use:

	Yes	No
ESPEN guidelines		
Scores (Nutric, NRS-2002)		
Weight		
BMI		

Other (specify)

* 13. How do you assess protein and calorie needs?

	Yes	No
Indirect calorimetry		
Equations (Es. Harris-Benedict)		
ESPEN guidelines suggestion (eg. 25 kcal/kg)		

Other (specify)

* 14. When do you start nutrition support from the moment in which the patient is clinically stable?

- 0-24 h
- 25-48 h
- Beyond 48 h

* 15. Which route of nutrition support do you use preferentially to reach the calorie and protein target?

- Parenteral
- Enteral
- Mixed

* 16. How much time do you take to reach the nutritional target set upon admission, for an average critically-ill patient?

	COVID-19	NON COVID-19
4-7 days	☐	☐
7-14 days	☐	☐
More than 14 days	☐	☐

* 17. Which of the following tools do you preferentially use to reach the nutritional targets in case of intolerance to enteral nutrition:

- Prokinetic drugs independently of the level of Gatsric residual volume (GRV)
- Prokinetic drugs with a threshold of GRV
- Supplementary parenteral nutrition
- Total parenteral nutrition
- Post-pyloric feeding
- Prokinetics and parenteral nutrition

* 18. How often do you perform the nitrogen balance in critically ill patients?

- On a daily basis for the whole duration of ICU stay
- On a daily basis for the first week
- Less than daily
- No

* 19. Which of the following enteral formulae are used in the first week of ICU stay in the critically-ill COVID-19 patient:

- ◯ Standard, isocaloric formula
- ◯ Individualized formula
- ◯ Not specified

* 20. Which of the following parenteral formulations are used in the first week of ICU stay in the critically-ill COVID-19 patient:

- ◯ Standard, commercially-available formulation
- ◯ Special formulation
- ◯ Formulation individually prescribed by a clinical nutrition specialist
- ◯ Not specified

* 21. Are nutritional problems reported in ICU the discharge summary?

- ◯ Yes
- ◯ No

* 22. What type of nutritional support is prescribed to patients after resolution of the acute phase of illness?

- ◯ Oral nutritional supplements
- ◯ Ongoing enteral nutrition
- ◯ Ongoing parenteral nutrition
- ◯ Not indicated

* 23. Do you use any validate scale for the functional assessment of critically ill patients?

- ◯ Yes
- ◯ No

If yes, please specify

[ ]

* 24. How do you assess lean body mass and monitor the nutritional staus of the critically ill patient?

- ◯ Bioimpedentiometry
- ◯ Muscle ultrasound
- ◯ Urinary nitrogen
- ◯ Other (specify)
