# Supplementary material for: Nutrition support for critically ill patients during the COVID-19 pandemic: the Italian SIAARTI survey
Source: J Anesth Analg Crit Care. 2022 Aug 9;2:35. doi: 10.1186/s44158-022-00063-6 (PMC9361260; doi:10.1186/s44158-022-00063-6)
Supplement: Supplementary file 2 — Additional file 2: Supplementary Figure S2. Relationship between nutritional risk scores, nutritional requirements and monitoring of the metabolic intervention and the type of Hospital of the respondents. Supplementary Figure S3. Relationship between the route of nutrition and time to start the support and to reach the targets and the type of Hospital of the respondents. Supplementary Figure S4. Relationship between the types and formulation of artificial support administered and the type of Hospital of the respondents. Supplementary Figure S5. Relationship between the strategies to overcome enteral nutrition intolerance and the type of Hospital of the respondents. [file 44158_2022_63_MOESM2_ESM.docx]

**Nutrition support for critically-ill patients during the COVID-19 pandemic: the Italian SIAARTI Survey**

**Short Title:** Nutritional support in critically-ill COVID-19 patients

### Romano Tetamo, Ciro Fittipaldi, Salvatore Buono, Michele Umbrello

**SUPPLEMENTARY APPENDIX**

**Supplementary figure S2. Relationship between nutritional risk scores, nutritional requirements and monitoring of the metabolic intervention and the type of Hospital of the respondents**

**
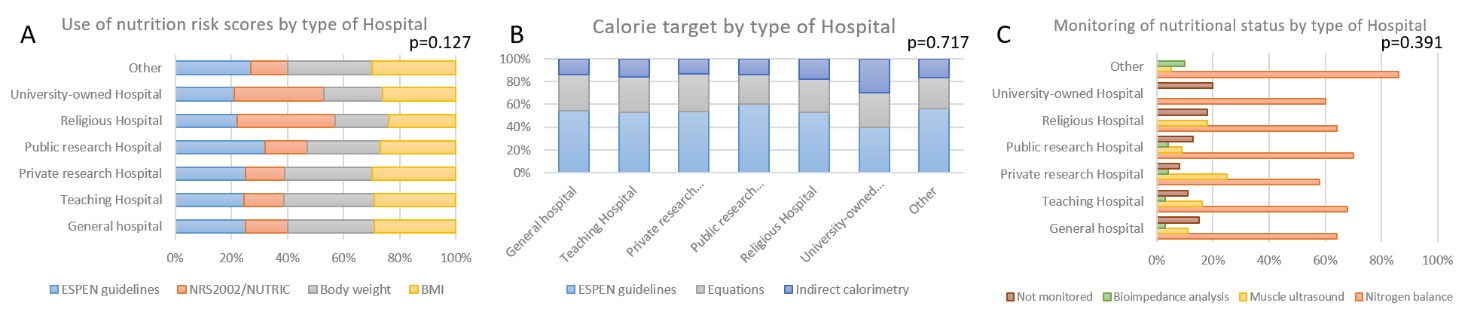
**

**Supplementary figure S3. Relationship between the route of nutrition and time to start the support and to reach the targets and the type of Hospital of the respondents.**

**
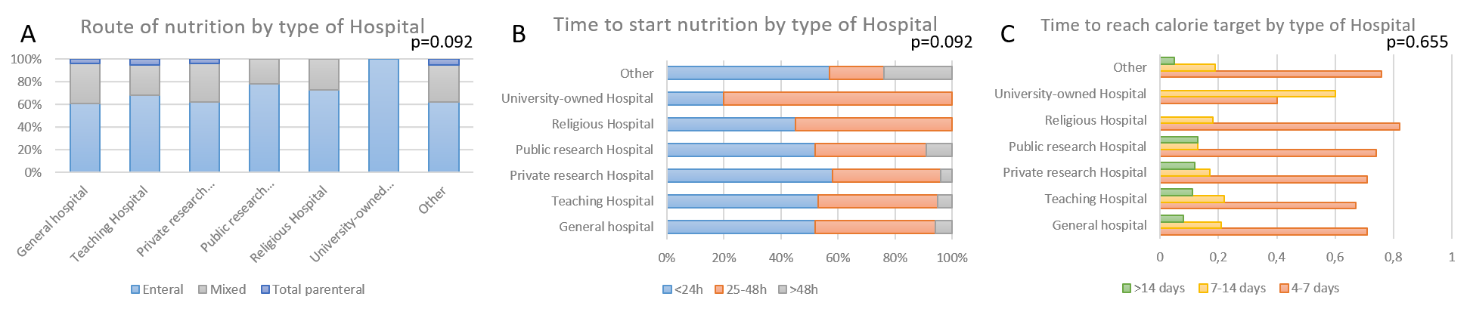
**

**Supplementary figure S4. Relationship between the types and formulation of artificial support administered and the type of Hospital of the respondents.**

**
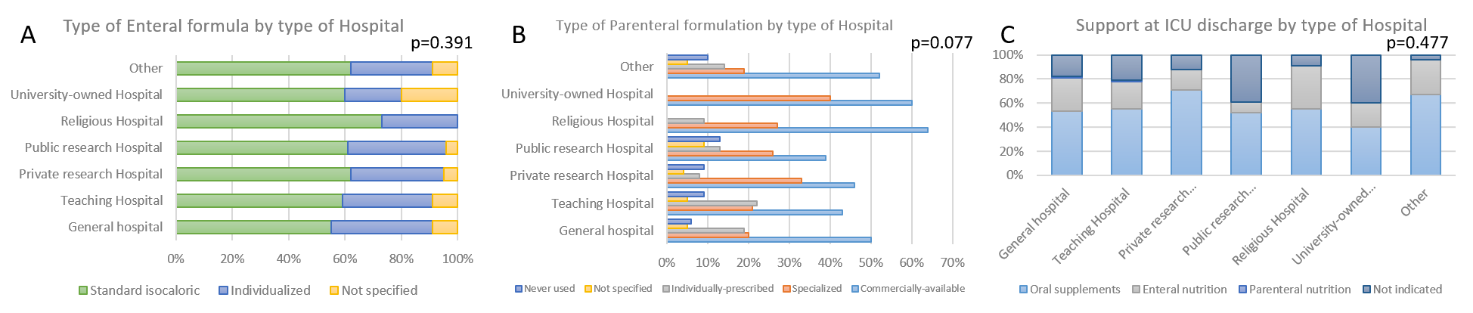
**

**Supplementary figure S5. Relationship between the strategies to overcome enteral nutrition intolerance and the type of Hospital of the respondents.**

**
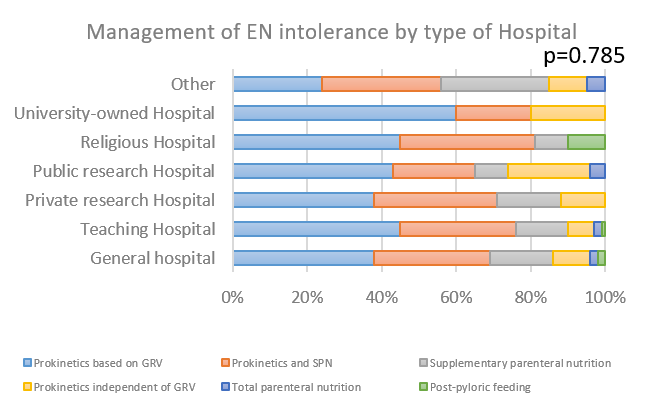
**
